# Application of Magnetically Assisted Reactors for Modulation of Growth and Pyocyanin Production by *Pseudomonas aeruginosa*

**DOI:** 10.3389/fbioe.2022.795871

**Published:** 2022-03-09

**Authors:** Joanna Jabłońska, Kamila Dubrowska, Adrian Augustyniak, Marian Kordas, Rafał Rakoczy

**Affiliations:** ^1^ Department of Chemical and Process Engineering, Faculty of Chemical Technology and Engineering, West Pomeranian University of Technology in Szczecin, Szczecin, Poland; ^2^ Chair of Building Materials and Construction Chemistry, Technische Universität Berlin, Berlin, Germany

**Keywords:** phenazines, bioprocessing, rotating electromagnetic field, static electromagnetic field, bacterial growth

## Abstract

*Pseudomonas aeruginosa* is a producer of desired secondary metabolites, including pyocyanin. Potential uses of this pigment urge a search for improved production methods. Recent trends in bioprocessing show the potential of the use of electromagnetic fields (EMFs) to influence the growth of microorganisms and even modulate the concentration of bioproducts. Here, we aimed at assessing the influence of rotating magnetic field (RMF) and static magnetic field (SMF) on pyocyanin production, growth rate, and respiration of *P. aeruginosa.* Moreover, exposure time to EMFs (2, 6, and 12 h) and culture volume (10 and 50 ml) were initially assessed. *P. aeruginosa* was cultivated in magnetically assisted reactors with 5 and 50 Hz RMF (magnetic induction of 24.32 and 42.64 mT, respectively) and SMF (−17.37 mT). Growth kinetics was assessed with Gompertz equation. The viability was tested using resazurin assay, whereas pyocyanin production by chloroform-HCl methodology. The growth of *P. aeruginosa* was slightly stimulated by exposure to a RMF with 50 Hz (108% related to the control) and significantly by SMF (132% related to the control), while RMF 5 Hz exposure prolonged the time of inflection (in comparison to RMF 50 Hz and SMF). The 6-h exposure to EMFs resulted in the highest pyocyanin production in comparison to the control, indicating a relationship between exposure time and product concentration. Moreover, cultures led in smaller volumes produced more pyocyanin. Our findings show that the use of different EMF types, frequency, and exposition time and volume could be used interchangeably to obtain different bioprocess aims.

## Introduction

*P. aeruginosa* is a bacterium of biotechnological and medical significance, mostly associated with wound infections and cystic fibrosis complications (Bassetti et al., 2018; Grygorcewicz et al., 2020). It is also well-known for the production of pigments, i.e., pyocyanin. This pigment exhibits unique properties that may find use in different branches of technology or medicine. It shows the potential to be applied in agriculture as an agent inducing systemic immunity in plants attacked by fungi and bacteria. Moreover, the anticancer activity of pyocyanin was previously reported (Moayedi et al., 2018). *P. aeruginosa* produces this pigment for electrochemical communication with other cells. Thanks to a high electron transfer capability, pyocyanin found use in colorimetric redox indices and was used as a component of the sensors or the OLED screens (Pierson and Pierson, 2010). Such chemical activity can also be used in microbial fuel cells to increase electric output (Feng et al., 2018). In addition, a recent discovery showed pyocyanin’s potential to induce prophage conversion (Jancheva and Thomas, 2021). In environmental biotechnology, pyocyanin can play a vital role in the biodegradation of petroleum-derived environmental contaminants (Viana et al., 2018). All shown applications indicate that demand for pyocyanin on the market may increase in the near future. Therefore, investigating novel methods to increase the pyocyanin production is justified and necessary.

Recent trends underline the potential of electromagnetic fields (EMFs) in assisting biological processes. Both static and alternating electromagnetic fields were previously reported to influence the growth and viability of various microorganisms, as well as their metabolism (Konopacki and Rakoczy, 2019; Piazzi et al., 2019; Kim et al., 2020; Masood et al., 2020; Jabłońska et al., 2021; Verburg et al., 2021). Xiong (Xiong et al., 2020) and Zhang (Zhang et al., 2015) reported increased pigments production by *Monascus purpureus* after exposure to a low-frequency magnetic field (50 Hz, 1.8, and 0.4 mT, respectively) ranging from around 40–70%. Deamici (Deamici et al., 2021) reported increased polysaccharide production while exposing microalgae to a static magnetic field (SMF). Raouia (Raouia et al., 2020) confirmed that SMF (200 mT) increased the amount of pyocyanin produced by *P. aeruginosa*. The literature analysis shows the possibility of the application of EMFs to enhance bioprocesses (Struk et al., 2017; Wasak et al., 2019). While SMF was initially tested in this direction, no work focused on an alternating current field such as a rotating magnetic field (RMF).

In this work, we aimed at investigating the hypothetical relationship between the exposition to rotating (5 and 50 Hz) and static magnetic fields and pyocyanin production in *P. aeruginosa*. An additional aim was to initially analyse the process parameters, considering the time of incubation, different volumes of the culture, and basic physiological measures such as maximum growth rate, inflection point and respiration.

## Methods

The strain used in the research was *Pseudomonas aeruginosa* ATCC^®^ 27,853^™^. Bacteria were stored at −20°C in trypticase soy broth medium (TSB) containing 10% v/v glycerol, revived prior to each experiment by plating on a trypticase soy agar medium (TSA) and cultivated at 37°C for 24 h. Overnight culture (from a single colony suspended in TSB) cultivated at 37°C was used for inoculations. Then, King A medium (1.64 g/L—MgCl_2_, 20.00 g/l—peptone, 10.00 g/L—K_2_SO_4,_ and 10 ml of glycerol per 990 ml of the medium) was inoculated with the overnight culture (1:100 v/v).

### Magnetically-Assisted Reactors and Field Characterization

**FIGURE 1:**
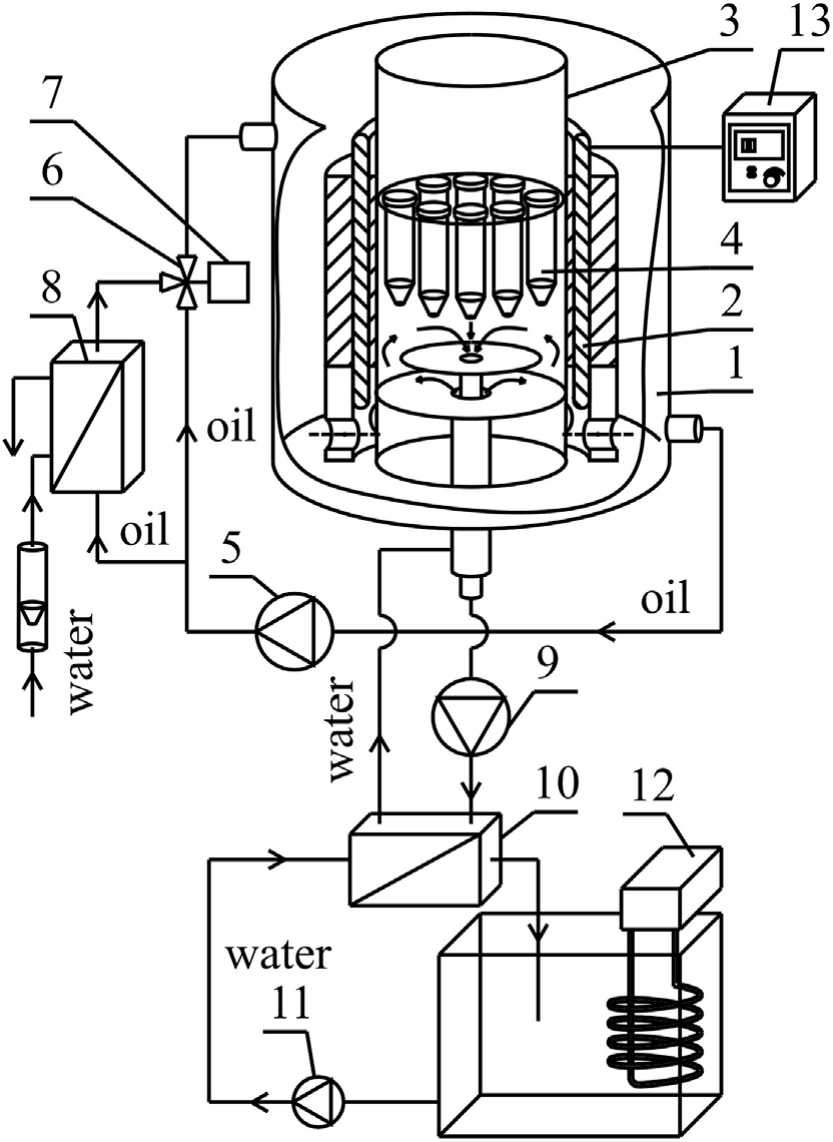
Simplified experimental set-up: 1—housing, 2—magnetic field generator, 3—cylindrical conduit, 4—probe, 5—circulating pump, 6—three-way valve, 7—actuator, 8—heat exchanger, 9—circulating pump, 10—heat exchanger, 11—circulating pump, 12—thermostat, 13—AC transistorized inverter, 14—DC power supply.

The illustrative experimental set-up is presented in Figure 1 and described in detail in Supplementary Materials (Supplementary Section S1.1, Supplementary Figure S1).

### Characterization of Electromagnetic Fields

The values of magnetic induction at different points inside the cylindrical conduit chamber were detected by the transverse Hall probe (STD18-0404) connected with the G meter (FW Bell 5,180 Magnetometer G meter; Magnetic Science Inc., United States) with the measurement accuracy equal to ± 2.5% of the reading. The measurements of the magnetic induction were carried out in the active area of the stator (“stator tooth”). The methodology of measurements was described in detail in Supplementary Materials (Supplementary Section S1.2, Supplementary Figures S2–S8).

The magnetic induction values obtained for RMF were equal to 24.32 and 42.64 mT for the frequency of electrical current *f* = 5 Hz and *f =*50 Hz, respectively. 5 and 50 Hz are the minimum and maximum values that can be obtained in constructed reactors, respectively. In the case of SMF, the investigations were carried out for the minimal value (−17.37 mT) of the magnetic field in the tested set-up.

### Growth Curves

The growth curves assay was conducted by incubation of the cultures in reactors for 12 h, with optical density (*λ* = 600 nm) and viability measurements (resazurin assay, *λ*_ex_ = 520 nm, *λ*_em_ = 590 nm) conducted every 2 h for 12 h. The experiment was conducted in four replicates (with eight repetitions within each sample).

The measurements were evaluated by performing Gompertz equation (Winsor, 1932) and calculating maximum growth rate (*µ*_max_) (Supplementary Materials, Supplementary Section S2.2).

The influence of the exposure to electromagnetic fields on the growth and viability was expressed by the growth index (*I*_growth_, Eq. 1) and viability index (*I*_viability,_ Eq. 2).

$$I_{growth}=\;\frac{{\left({µ}_{\max}\right)}_{field}}{{\left({µ}_{\max}\right)}_{control}}\;\times 100\%$$ (1)

$$I_{viability}=\;\frac{{\left(viability\right)}_{field}}{{\left(viability\right)}_{control}}\;\times 100\%$$ (2)

where:

$I_{growth}$ —growth index, %

$I_{viability}$ —viability index, %

${\left(\mu_{\max}\right)}_{field}$ – maximum growth rate under the action of rotating or static magnetic field, h^−1^

${\left(\mu_{\max}\right)}_{control}$ —maximum growth rate for control (without the action of rotating or static magnetic field effect), h^−1^

${\left(viability\right)}_{field}$ —viability under the action of rotating or static magnetic field

${\left(viability\right)}_{control}$ – viability under the action of rotating or static magnetic field

### Pyocyanin Quantification

Cultures prepared in Falcon-type tubes in volumes of 10 and 50 ml were placed in the magnetically assisted reactors and withdrawn after 2, 6, or 12 h of the exposure to MF and further incubated (37°C) to reach 48 h. Pyocyanin concentration was quantified exploiting chloroform-HCl methodology (Essar et al., 1990). Shortly, a 5 ml sample was collected from centrifuged culture and mixed with 3 ml of chloroform (1 h on roller shaker). The chloroform phase was collected and mixed with 1 ml of 0.2N HCl. Samples were vortexed for 30 s, the acid phase was collected, and absorbance was measured at *λ* = 520 nm. The experiment was conducted in three separate replicates. The concentration was calculated employing the calibration curve (Supplementary Figure S9).

The results are presented in comparison to the control culture (unexposed to the electromagnetic field) and expressed as pyocyanin production index (*I*_PYO_, Eq. 3).

$$I_{PYO}=\;\frac{{\left(Y_{PYO}\right)}_{field}}{{\left(Y_{PYO}\right)}_{control}}\;\times 100\%$$ (3)

where:

$I_{PYO}$ —pyocyanin production index, %

${\left(Y_{PYO}\right)}_{field}$ – pyocyanin concentration under the action of rotating or static magnetic field, µg/ml

${\left(Y_{PYO}\right)}_{control}$ – pyocyanin concentration without the action of rotating or static magnetic field, µg/ml

## Results

### Growth Kinetics

**FIGURE 2:**
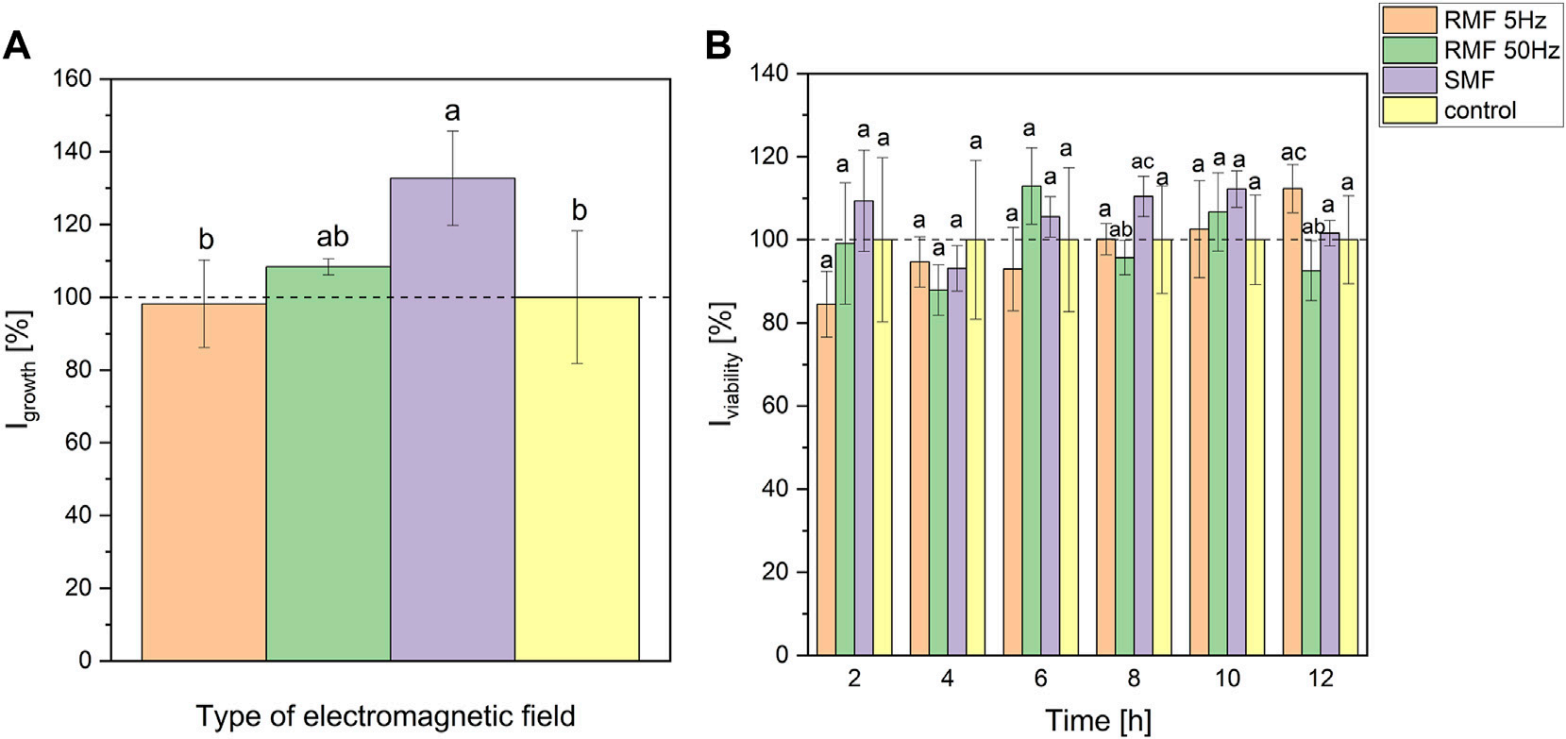
The influence of SMF and RMF on growth index **(A)** and viability index **(B)** of *P. aeruginosa*. The error bars indicate the relative standard error of the mean. Superscripts with different letters are considered statistically different at *p* < 0.2 for **(A)** and at *p* < 0.1 for **(B)**.

Figure 2A presents the results of the maximum growth rate suggesting that static and rotating magnetic fields (50 Hz) stimulated the growth of *P. aeruginosa* by around 32 and 8% (on average), respectively. However, statistically significant differences occurred only between SMF and control, and RMF 5 Hz and SMF (at *p* < 0.2). Parameters resulting from the approximation with Gompertz equation and box plots are presented in Supplementary Materials (Supplementary Section S2.1, S2.2, Supplementary Figures S10, 11). The viability of cells during exposure to electromagnetic fields depended on the type of EF and its frequency (in the case of RMF) (Figure 2B). At the beginning of cultivation, 5 Hz RMF led to a slight decrease in *P. aeruginosa* viability. However, the viability was slowly increasing over the incubation time, reaching a higher value than the control culture after 12-h incubation. The viability of *P. aeruginosa* exposed to rotating magnetic field with 50 Hz and static magnetic field was changing irregularly. Nevertheless, it is worth underlining that tested cultures exhibited higher viability (respiration rate) in the sixth hour while incubated with RMF 50 Hz, and 2nd, 6th, 8th, and 10th hour for SMF (Figure 2B). However, one-way ANOVA analysis (at *p* < 0.1) showed significant differences in resazurin assay only for RMF 50 Hz and SMF cultures at 8th hour (95 and 110% related to the control, respectively), and RMF 50 Hz and RMF 5 Hz at 12th hour of incubation (93 and 112% related to the control, respectively).

Moreover, the inflection point parameters (time and optical density at inflection point) were calculated and expressed as I_infl_. The methodology and results are presented in Supplementary Materials, Supplementary Section S2.2 (Supplementary Figure S12). Statistical analysis (*p* < 0.1) revealed that time of inflection differed for RMF 5 Hz and RMF 50 Hz (107 and 94% related to the control, respectively), and RMF 5 Hz and SMF (107 and 95% related to the control, respectively). At the same time, OD did not differ in any tested setups (at *p* < 0.2).

### Pyocyanin Quantification

**FIGURE 3:**
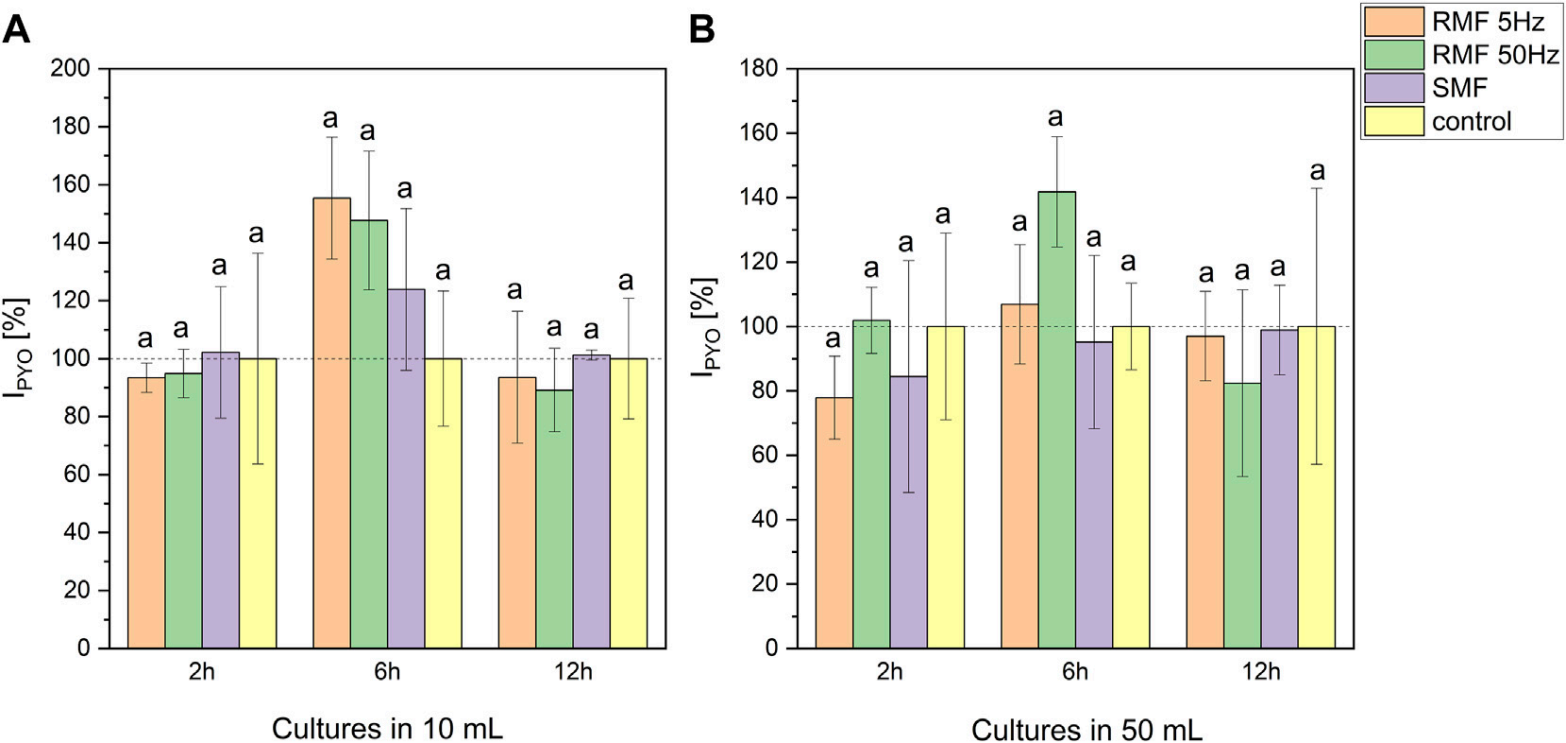
The influence of SMF and RMF on pyocyanin production index by *P. aeruginosa* in 10 ml **(A)** and 50 ml **(B)** cultures. The error bars indicate the relative standard error of the mean. Superscripts with different letters are considered statistically different at *p* < 0.2.

Pyocyanin production under EMF exposition was diversified (Figure 3). The volume of the culture influenced the concentration. Cultures cultivated in 10 ml produced on average more pigment than cultures led in 50 ml (33.20–52.93 and 8.19–13.54 μg/5 ml, respectively). ANOVA analysis (Supplementary Figure S13) revealed significant differences at 6th and 12th hour of incubation in all tested setups. Moreover, the highest production of the pigment in comparison to the control culture was noted after 6-h-long incubation in the electromagnetic field (Figure 3). This tendency was detected in all tested EMF types in 10 ml cultures (155% for RMF 5 Hz, 148% for RMF 50 Hz and 124% for SMF related to the control) and RMF 50 Hz in 50 ml cultures (142% in relation to the control). Even though the trend showed that 6-hour-long exposure enhanced pyocyanin production, these results did not significantly differ from control cultures at *p* < 0.2.

The effect of exposure to the electromagnetic fields differed based on the applied field type or its frequency. Overall, SMF exposure increased the maximum growth rate when compared to the control. However, pyocyanin production, in that case, was the lowest among tested electromagnetic fields. The 50 Hz RMF slightly enhanced the growth rate and led to elevated pyocyanin production when compared to the control. 5 Hz RMF exposure did not affect the growth rate of *P. aeruginosa* but resulted in a higher pyocyanin productionin the case of 10 ml cultures (Figure 3A). At the same time, the time at inflection point was significantly longer for RMF 5 Hz than for RMF 50 Hz and SMF. The incubation in EMF reduced the viability in the case of RMF 50 Hz at the stationary phase. However, the cell viability was increased at certain time points for SMF and RMF 5 Hz. The observed elevated viability of SMF-exposed *P. aeruginosa* was following the increased growth rate in cultures.

## Discussion

It was previously suggested that electromagnetic fields (EMFs) could repeatably influence the growth and viability of microorganisms (Tessaro et al., 2015; Oncul et al., 2016; Konopacki and Rakoczy, 2019). Following previous studies, we hypothesized that there might also be a relationship between the exposition to EMF and pyocyanin production in *P. aeruginosa* that has been explored to date by one research group that assessed the influence of SMF 200 mT and showed an increase in pyocyanin production (Raouia et al., 2020). Our results concerning SMF cover the magnetic induction of −17.37 mT and did not show very pronounced effect on pigment production, hence it may be hypothesized that higher magnetic induction can result in higher pyocyanin concentration. Previous works dealing with the influence of UV and gamma radiation also reported enhanced pigment production by *Serratia marcescens* (El-Bialy and Abou El-Nour, 2015; Elkenawy et al., 2017).

We confirmed the modifying effect of different EMFs on the growth rate, viability, and pigment production, making the use of EMF an interesting option in bioprocess engineering. Furthermore, the presented outcome shows that the use of a particular EMF might be selected for a given purpose rather than a general positive/negative influence on bacteria suggested in other sources (Oncul et al., 2016; Konopacki and Rakoczy, 2019). In the case of *P. aeruginosa,* the focus on faster growth of the culture would imply the use of SMF rather than RMF, while pyocyanin production could possibly be increased by applying 50 Hz RMF. Furthermore, we have shown that the increased growth rate (as in the sample exposed to SMF) does not need to result in the clearly upregulated production of substances of interest.

Although the mechanisms behind the observed changes are suspected, there is still no clear explanation. It was hypothesized that EMF might generate an excess of reactive oxygen species (ROS) that may be debilitating for cells causing their death (Wang and Zhang, 2017). However, microorganisms have mechanisms for diminishing their effect involving peroxidases or glutathione. Lemire et al. (2017) indicated that in some cases, oxidative stress could even increase energy production in bacteria, e.g., leading to overproduction of NADPH. This could suggest increased viability detected in resazurin assay in some cases, particularly in samples exposed to SMF (Wang and Zhang, 2017). The differences observed between the two types of EMF could be explained by their different characteristics.

Rogers and Hore (Rodgers and Hore, 2009) and Anton-Leberre et al. (2010) point out that SMF can alter the energy levels of certain molecules, but the application of this kind of magnetic field does not cause field energy transmission to the living cells. The alternating magnetic fields (such as RMF) can generate eddy currents that induce a current within the medium, produce small dynamos and lead to micromixing of the culture, where the increase in frequency strengthens this phenomenon (Konopacka et al., 2019). These eddy currents may potentially increase the availability of oxygen via increasing its dispersion in the growth medium (Rakoczy et al., 2017). Pyocyanin production depends on oxygen availability as it was shown that oxygen carriers such as n-hexane could result in an increased release of the pigment (Ozdal et al., 2019). We suspect that this could be the cause of the highest pyocyanin content observed in samples.

Shifts in membrane potential caused by EMF could be another mechanism influencing the growth rate and respiration of studied bacteria. Oncul et al. (2016) reported changes in bacterial membrane polarization in *E. coli* and *S. aureus* exposed to the low-frequency electromagnetic field. Similar to some of our findings, the authors have recorded increased respiratory activity.

With current results, we also show another aspect that was not previously elucidated in the literature. The exposition time to EMF may also play a role in the pyocyanin production process using *P. aeruginosa*. We have shown that samples incubated in the magnetically assisted bioreactor for 6 h resulted in highest pyocyanin concentration in comparison to the control. We hypothesise that it may be connected to the phase of bacterial growth as after 6 h the culture reaches the late logarithmic or early stationary phase in the studied conditions. Previous research reports showed that pyocyanin production starts around this time of the culture (Gonçalves and Vasconcelos, 2021). So far, most of the works either exposed the samples to EMF during the total experiment time or used arbitrarily selected time points (Struk et al., 2017; Konopacki and Rakoczy, 2019). Our finding indicates a possible relationship between the time of exposure and the outcome that may be useful for designing magnetically assisted bioprocesses. Moreover, obtained results show the importance of the culture volume and proved that smaller volumes resulted in higher pyocyanin concentration. We hypothesize that *P. aeruginosa* may produce pyocyanin only in the interphase area (where the oxygen availability is high).

## Conclusion

Based on the conducted research, we report that both static and rotating electromagnetic fields may play a role in the modulation of *P. aeruginosa* physiology, e.g., growth rate, respiration, or pyocyanin production. Moreover, it is the first report of the influence of RMF on pyocyanin production. Furthermore, the type of EMF, the frequency (in RMF), culture volume and the exposition time may specifically influence bacteria. These results show that modifying the conditions of magnetically assisted bioprocesses can bring different outcomes such as faster growth of the culture or desired secondary metabolites (e.g., pyocyanin).

Nevertheless, the molecular mechanisms behind the observed effects remain unclear. This underlines the necessity to further optimize the process conditions and run more thorough analyses concerning magnetic induction value, exposure time, field type, and culture volume.

## Data Availability

The raw data supporting the conclusion of this article will be made available by the authors, without undue reservation.
