# Supplementary material for: Application of Magnetically Assisted Reactors for Modulation of Growth and Pyocyanin Production by Pseudomonas aeruginosa
Source: Front Bioeng Biotechnol. 2022 Mar 9;10:795871. doi: 10.3389/fbioe.2022.795871 (PMC8959660; doi:10.3389/fbioe.2022.795871)
Supplement: Supplementary file 1 [file DataSheet1.pdf]

Supplementary Material

Supplementary material for Materials and methods section

1.1 Magnetically-assisted reactors

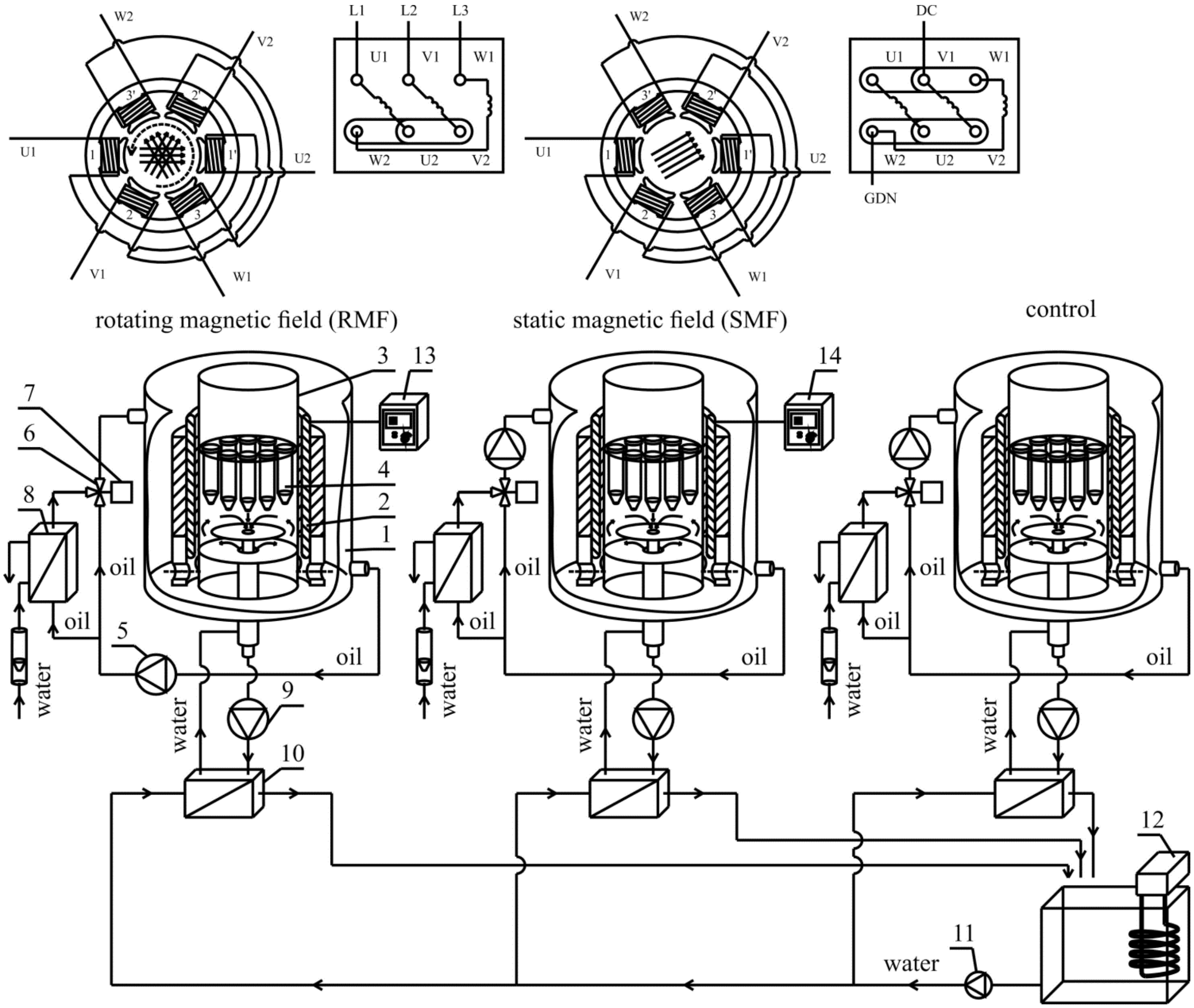

Figure S1. Experimental setup

The experimental set-up consisted of three reactors equipped with magnetic field generators, which can work separately. In the case of this work, one of the reactors was operated with the active generator of the rotating magnetic field (RMF). The generator of the second reactor was connected with the DC power supply. This reactor was used to produce the stationary magnetic field (SMF). The third reactor (control reactor) was used as the control apparatus (this generator was not connected to the current).

The RMF reactor consisted of the housing (1) and the generator of RMF (2). This reactor was constructed sing a three-phase stator of an induction squirrel cage motor. The RMF source was the stator windings powered by a balanced 3-phase AC power supply. This kind of MF has a constant intensity over time, while it changes its direction continuously at any point in the domain. The cylindrical conduit (3) was placed coaxially inside the generator of RMF (2). The AC transistorized inverter (13) was used to adjust the power supply frequency and the RMF frequency in the range between 5 and 50 Hz. The probes (4) with the culture medium were placed in the generator of the magnetic field.

The SMF reactor was connected with the DC power supply (14). The usage of this power supply and the connection of the stator windings (the diagram is shown in Fig. S1) was allowed to generate the SMF. The electric current, constant with time, generated this kind of magnetic field.

The space between the inner wall of the housing (1) and the outer wall of the cylindrical conduit (3) was filled with silicone oil. This liquid was used to remove the heat produced during the work of generators of the magnetic field. The circulation pump (5), the three-way valve (6) assembled with the actuator (7), and the heat exchanger (8) was used to remove the accumulated heat in the experimental apparatus.

To stabilize the temperature inside the reactor's process chamber, an additional temperature control system was used. The probes (4) were placed in a water bath with a constant temperature of 37 °C. The circulation pump (9) was used to force the water flow between the chamber of the reactor and the heat exchanger (10). The working liquid (water circulating between the reactor and the heat exchanger) was heated by the water from the thermostat (13) to establish the stable temperature of the liquid inside the rector (37±0.1°C).

1.2 Field characterization

The calibration of the probe has been done with the usage of a zero lux chamber (B = 0 mT). This probe has been mounted in the reactor with the generator of the magnetic field. A sketch of measurements points (marked as +) inside the reactor is showed in Fig. S2.

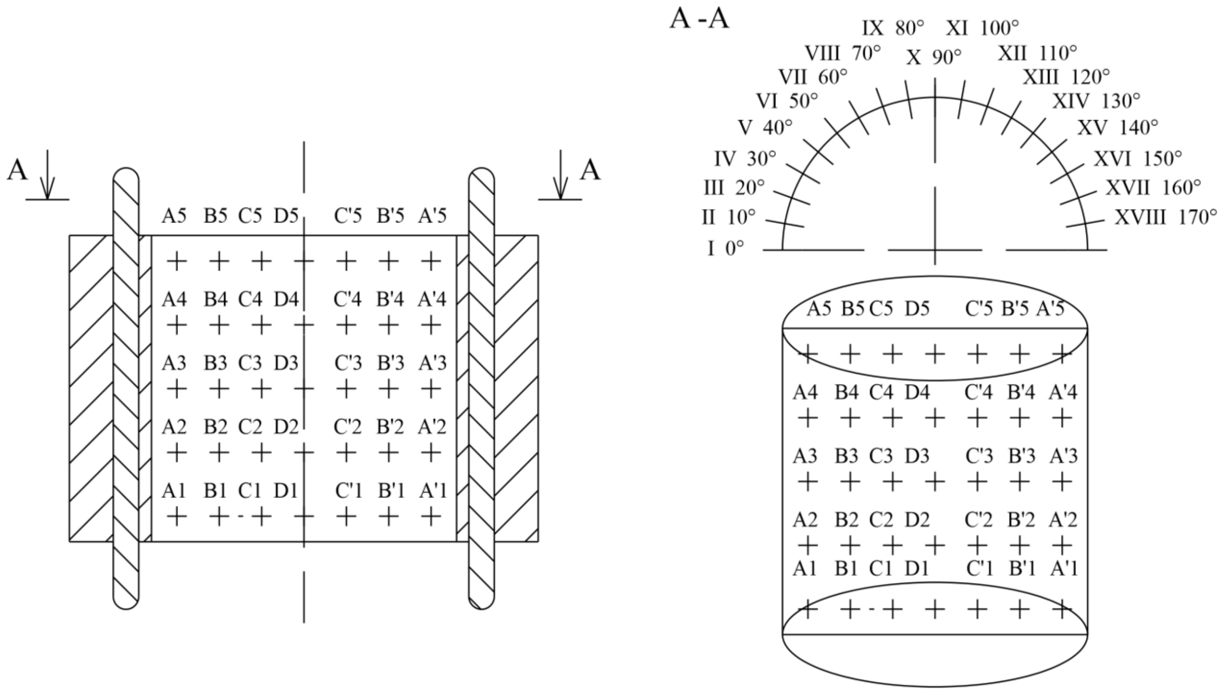

Figure S2. The sketch of measurement points of magnetic induction (marked as +) inside the reactor with the generator of the magnetic field. At these points, the changes in magnetic induction were measured.

The value of magnetic induction at each sampling point was calculated based on the records of the magnetic induction signals (the variety of this parameter over time). These calculations allowed us to determine the magnetic field patterns in the axial section of the magnetic field generator (these axial sections are marked in Roman numerals in Supplementary Figure 2.).

In the case of these investigations, the RMF is generated by coils located around the cylinder. Therefore, the additional measurements of the magnetic induction profiles for the various angular coordinate were carried out. The graphical presentation of the axial section in which the magnetic induction was measured is shown in Supplementary Figure 2. The grid of measurement points shown in Fig. S1 was applied to obtain the variation of magnetic induction for these axial sections.

1.2.1 Characterization of rotating magnetic field (RMF)

The typical records of the magnetic induction values versus time are presented in Supplementary Figure 3.

a) b)

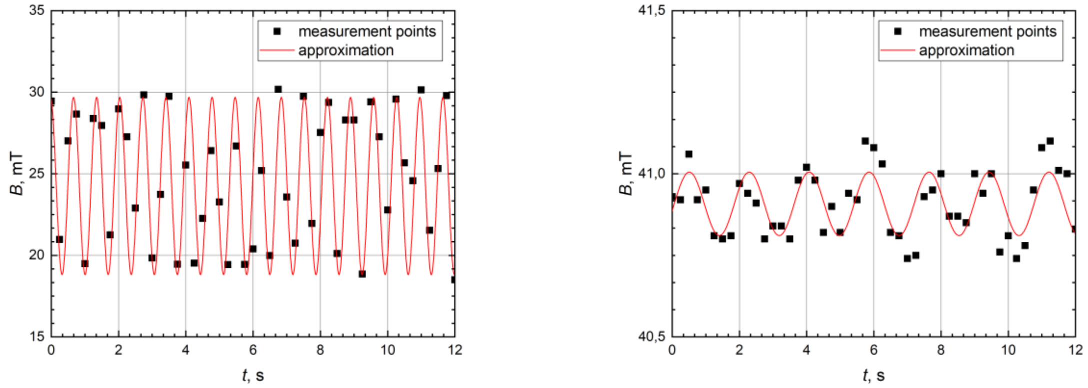

Figure S3. The typical example of the variation between recorded magnetic induction (*B*) and the time for the selected measurement point (point A3, see Fig. S1) inside the RMF reactor and the frequency of the electrical current *f* = 5 Hz (a) and *f* =50 Hz (b)

The following relationship is used to describe the scatter of the measurement points

$$B(t) = y_0 + A\sin\left[\frac{\pi(t - x_c)}{w}\right] \qquad \text{(S1)}$$

where:

B - magnetic induction, mT;

t - time, s;

y_0, A, x_c, w - parameters.

The variation of the magnetic induction values, *B*, over time, *t*, is mathematically defined for *f* = 5 Hz and *f* =50 Hz, respectively

$$B(t) = 24.25 + 5.43\sin\left[\frac{\pi(t - 0.49)}{0.34}\right] \qquad \text{(S2)}$$

$$B(t) = 40.91 + 0.10\sin\left[\frac{\pi(t-0.06)}{0.89}\right] \tag{S3}$$

Next, the averaged value for each measurement and the sampling point was calculated as follows

$$\overline{B} = \frac{1}{t}\int_0^t B(t)\,dt \tag{S4}$$

where:

$\overline{B}$ - the averaged value of magnetic induction at each sampling point (see Supplementary Figure 2) over time, mT

The calculated averaged values of magnetic induction at different points inside the RMF generator may be presented in the form of patterns of magnetic induction. Figure S4 shows the typical example of the contour patterns of the spatial distribution of the magnetic field in the selected axial sections of the RMF reactor (axial-section no. I; see Supplementary Figure 2).

a) b)

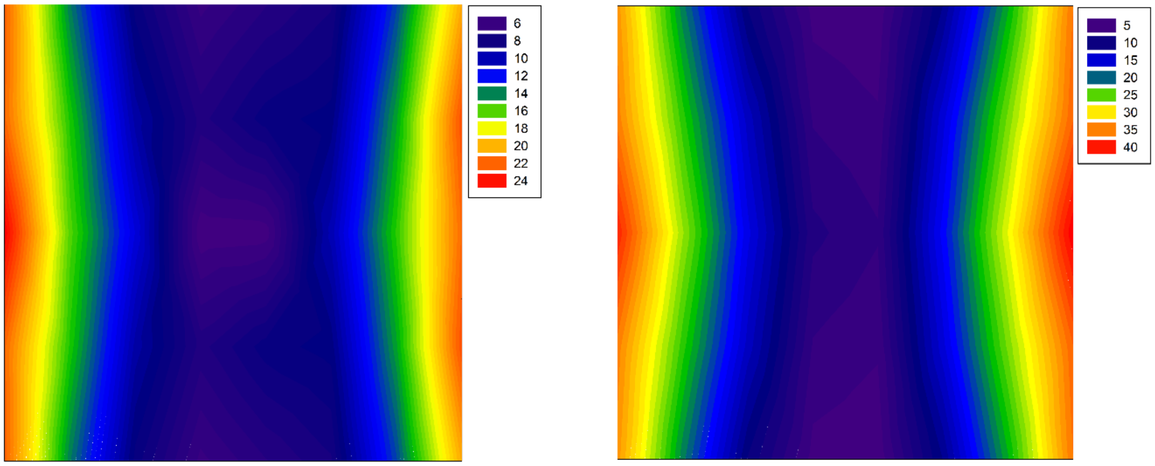

Figure S4. The typical example of the contour patterns of the spatial distribution of the magnetic field in the selected axial-section (no. I; Fig. S1) for the frequency of electrical current *f* = 5 Hz (a) and *f* =50 Hz (b).

The results obtained from the preliminary analysis of the iso-contour patterns of the spatial distribution of the magnetic induction are shown that the magnetic field is axially symmetric. Therefore, the magnetic induction does not depend on the angle *φ*, and the magnetic induction does

not vary with this spatial coordinate. As shown in Fig. S4, the maximal values of the magnetic induction were measured in the middle of the middle part of the RMF reactor. The obtained values of the magnetic induction may also be presented in the system of the polar coordinate system. The examples of the magnetic induction patterns in the middle part experimental set-up (cross-section of the RMF generator) are presented in Fig. Supplementary Figure 5.

a) b)

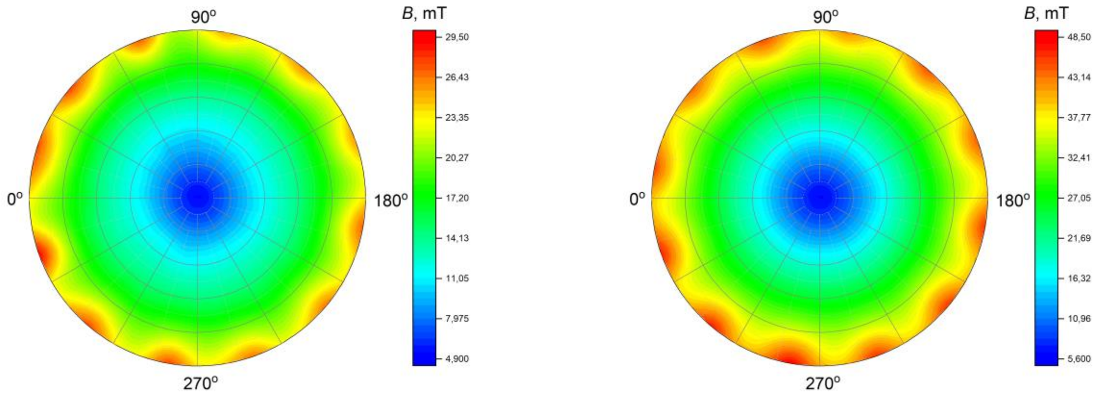

Figure S5. The contour patterns of the spatial distribution of the magnetic field in the selected cross-section of the RMF reactor for the frequency of electrical current *f* = 5 Hz (a) and *f* =50 Hz (b).

The probes with culture medium were placed in the area with the maximum values of the magnetic induction (see Supplementary Figure 6).

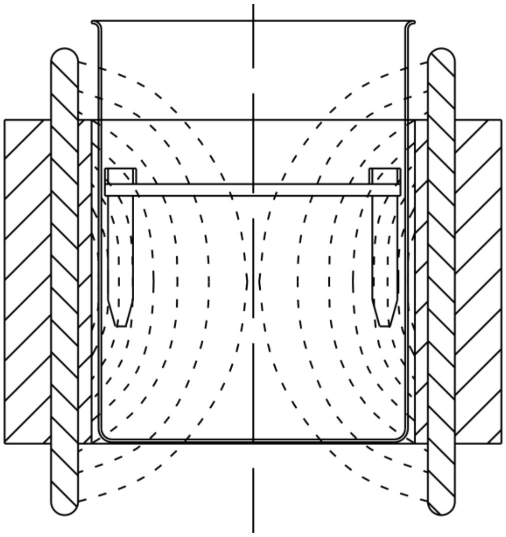

Figure S6. The localization of the probes with the culture medium inside the RMF reactor

1.2.2 Characterization of stationary magnetic field (SMF)

The values of the magnetic induction were measured at selected points inside the SMF reactor (see Fig. S2). The typical example of the magnetic induction patterns for this kind of magnetic field is presented in Supplementary Figure 7.

a) b)

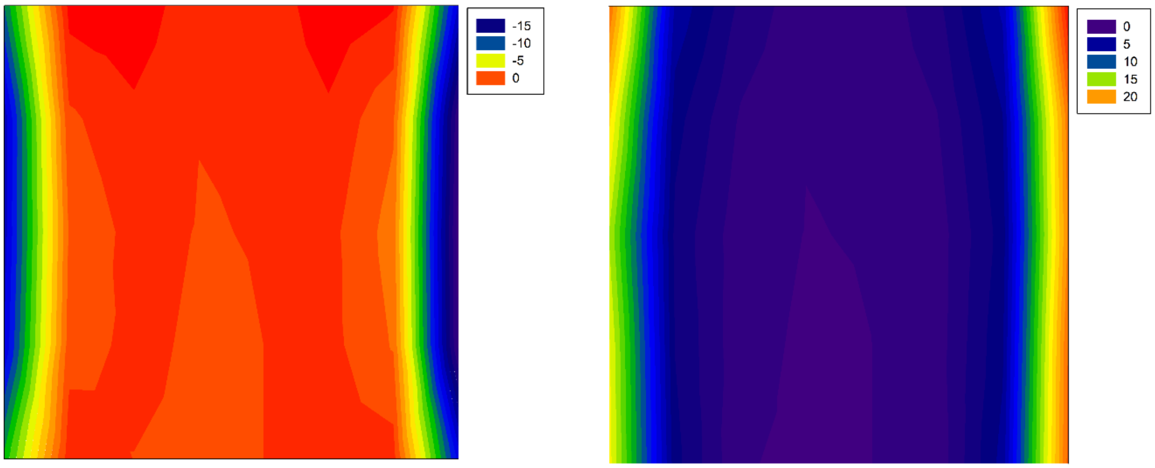

Figure S7. The typical example of the contour patterns of the spatial distribution of the magnetic field in the selected axial-section no. I (a) and no. IV (b) (see Fig. S2).

It should be noticed that the SMF has the minimum and maximum values in the middle of the SMF reactor. The values of the magnetic field were changed near the wall of the cylindrical conduit. Moreover, these values decreased up to 0 mT toward the center of the SMF reactor. Based on the experimental measurements, the minimal and maximal values of the magnetic induction were obtained. The variation of these values versus the number of the axial-section of the SMF reactor is graphically presented in Supplementary Figure 8.

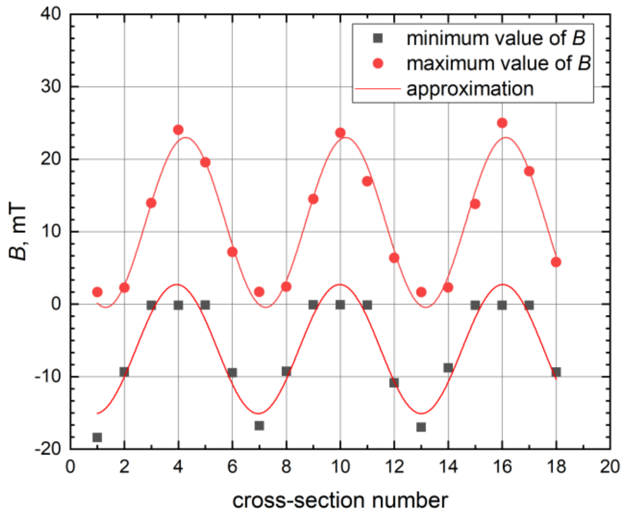

Figure S8. The graphical presentation of the relation between the minimal and maximal values of the magnetic induction and the axial-section number of the SMF reactor.

In the case of these measurements, the probes with the culture media were placed in the places where the minimum values of the SMF were recorded.

1.3 Calibration curve of pyocyanin concentrations

Calibration curve was prepared using pyocyanin concentrations of 1, 5 ,10, 20, 50, 100 and 1000 μg/mL dissolved in 0.2N HCl solution. The absorbance of each sample was measured at 520 nm and used to create a calibration curve (Fig. S9) that resulted in equation:

$$\text{PYO concentration} = 418.31 \times \text{Absorbance} - 19.41 \quad \text{(S5)}$$

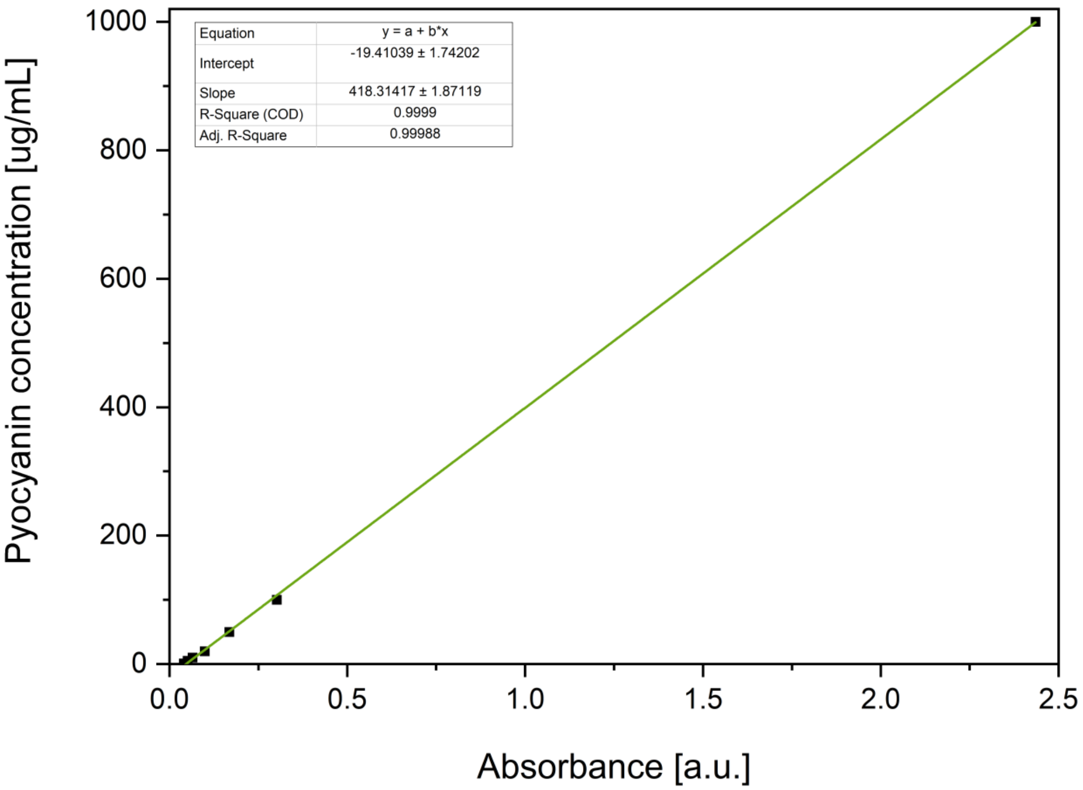

Figure S9. Calibration curve of different pyocyanin concentrations

Supplementary materials for the Results section

2.1 Box plots

Box plots of optical density measurements over 12 hours of culture, with or without exposure to electromagnetic fields, are presented in Supplementary Figure 10. The box presents both the median and mean values of the measurements. It is noticeable that the 4th hour of the culture was characterized with the biggest width in all presented cases. Such a result may be justified by the fact that this time was identified as the logarithmic phase.

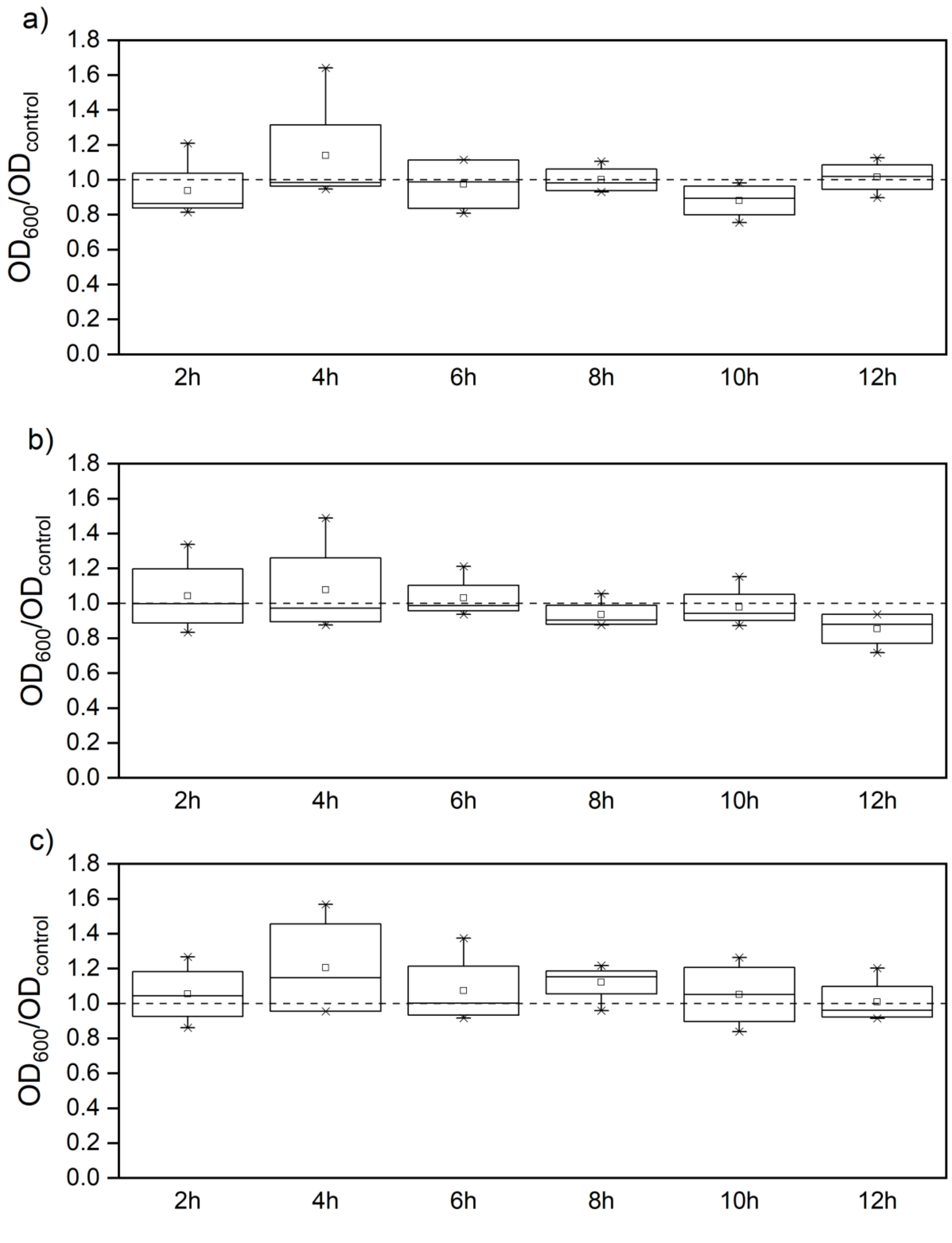

Figure S10. Box plots of optical density measurements of cultures exposed to a) RMF 5 Hz, b) RMF 50 Hz, c) SMF (4 separate repetitions, values are compared to control value in the corresponding experiment).

2.2 Exemplary Gompertz approximation

The Gompertz equation was used to assess the growth of bacteria and to calculate the growth parameters based on the optical density measurements. The results were presented as a growth parameter according to the equation

$$y = k \exp\left[-\exp\left(a - b x\right)\right] \tag{S6}$$

where:

y – optical density at a wavelength of 600 nm

a, b, k – parameters of Gompertz equation

x – time, h

e – Euler constant

To obtain parameters a, b and k, the optical density measurements were implemented in Origin 2021 Software. The data was assigned to the non-linear curve fit employing Gompertz approximation. Exemplary fitting was presented in Supplementary Figure 11.

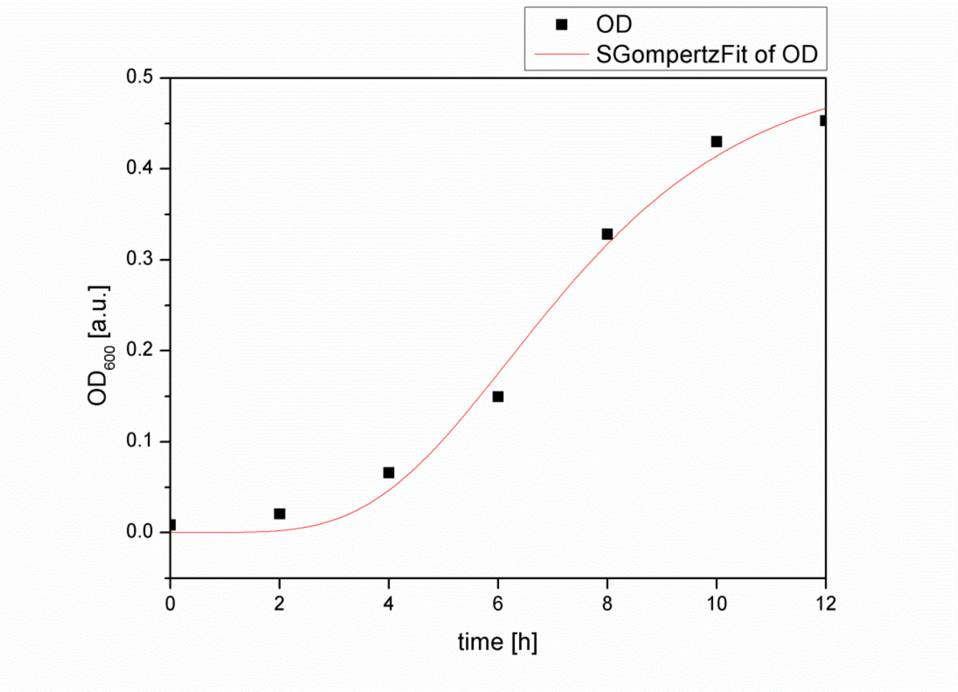

Figure S11. Example of Gompertz approximation based on optical density measurements

The fitting allowed to obtain a, b, and k parameters that were implemented for further calculations. The maximum growth rate was calculated according to

$$\mu_{\max} = \frac{bk}{e} \quad . \tag{S7}$$

Another parameter, the inflection point, was calculated using equation S8:

$$\begin{cases} t = \frac{a}{b} \\ OD = \frac{k}{e} \end{cases} \tag{S8}$$

where:

t – time of inflection [h]

OD – optical density of the culture at inflection time [a.u.]

Inflection parameters were calculated and then expressed as inflection point index:

$$I_{infl} = \frac{t_{field}}{t_{control}} \times 100\% \tag{S9}$$

and

$$I_{infl} = \frac{OD_{field}}{OD_{control}} \times 100\% \tag{S10}$$

Obtained results of the calculated inflection points are presented in Fig. S12.

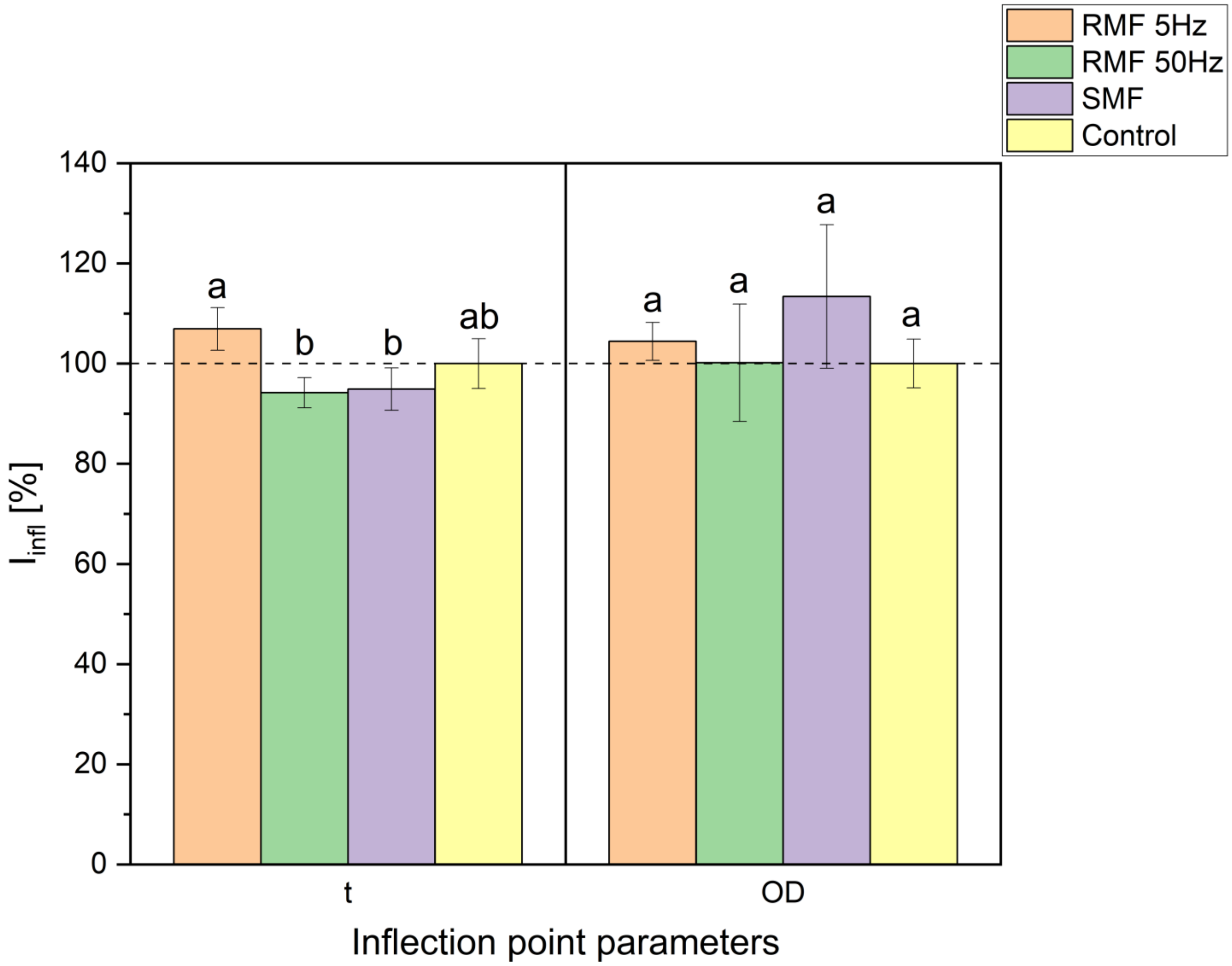

Figure S12. Inflection point parameters of the cultures exposed to electromagnetic field. Error bars indicate relative standard error of the mean. Superscripts with different letters are considered statistically different at $p<0.1$.

2.3 Pyocyanin production

The influence of the culture volume on pyocyanin production (expressed per 5 mL of the culture) is presented in Fig. S13.

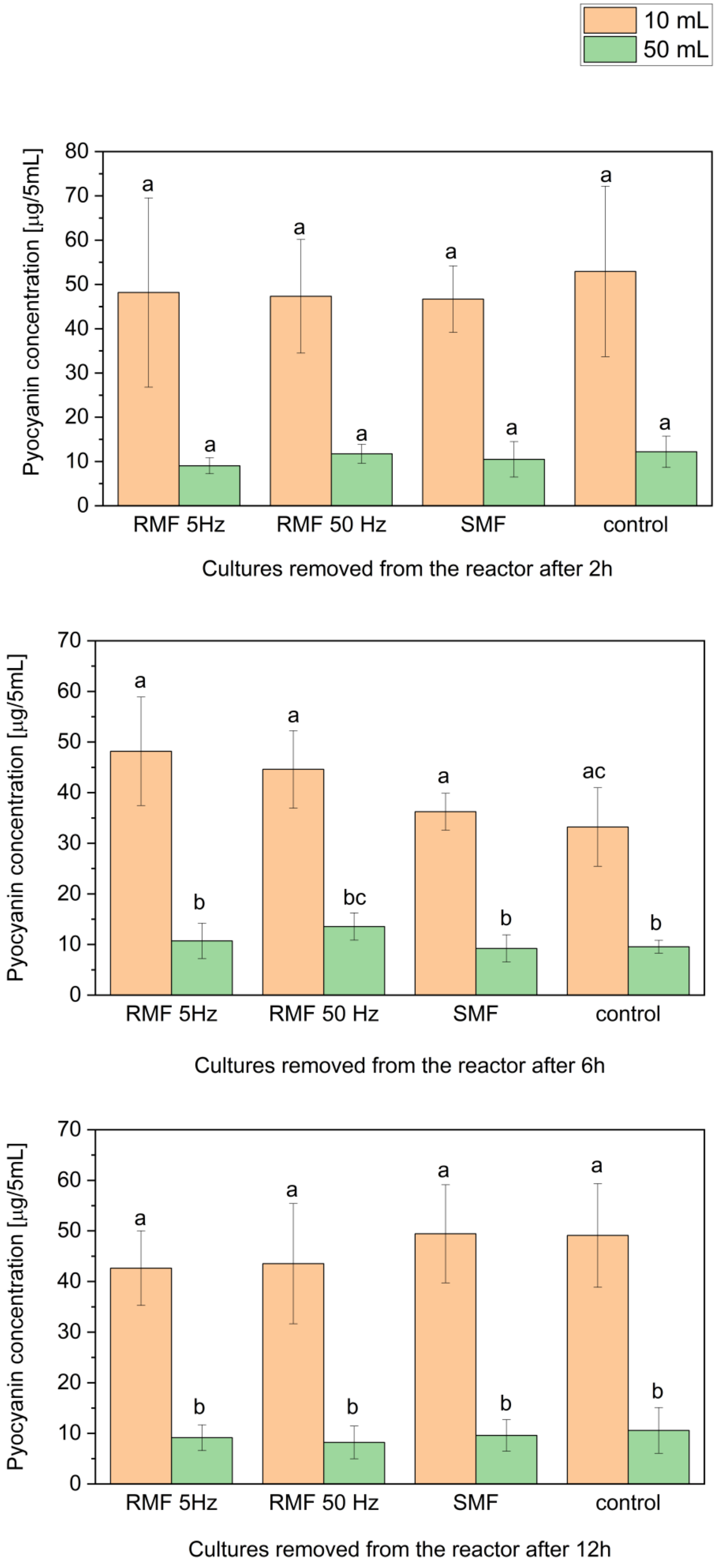
10 mL
50 mL
Pyocyanin concentration [µg/5mL]
RMF 5Hz
RMF 50 Hz
SMF
control
Cultures removed from the reactor after 2h
Pyocyanin concentration [µg/5mL]
RMF 5Hz
RMF 50 Hz
SMF
control
Cultures removed from the reactor after 6h
Pyocyanin concentration [µg/5mL]
RMF 5Hz
RMF 50 Hz
SMF
control
Cultures removed from the reactor after 12h

Figure S13. Comparison of different volumes of the cultures exposed to electromagnetic field. Error bars indicate standard error of the mean. Superscripts with different letters are considered statistically different at $p<0.2$.
